# Management of Venous Thromboembolism in Patients with Hereditary Antithrombin Deficiency and Pregnancy: Case Report and Review of the Literature

**DOI:** 10.1155/2017/9261351

**Published:** 2017-01-10

**Authors:** Mohammad Refaei, Lydia Xing, Wendy Lim, Mark Crowther, Kochawan Boonyawat

**Affiliations:** ^1^Division of Core Internal Medicine, Department of Medicine, University of Alberta, Edmonton, AB, Canada; ^2^Division of Hematology and Thromboembolism, Department of Medicine, McMaster University, Hamilton, ON, Canada; ^3^Department of Pathology and Molecular Medicine, McMaster University, Hamilton, ON, Canada; ^4^Department of Medicine, Ramathibodi Hospital, Mahidol University, Bangkok, Thailand

## Abstract

*Background*. Hereditary antithrombin deficiency is a thrombogenic disorder associated with a 50–90% lifetime risk of venous thromboembolism (VTE), which is increased during pregnancy and the puerperium in these patients. We present a case of a woman with antithrombin (AT) deficiency who presented with a VTE despite therapeutic low molecular weight heparin (LMWH). Though the pregnancy was deemed unviable, further maternal complications were mitigated through the combined use of therapeutic anticoagulation and plasma-derived antithrombin concentrate infusions to normalize her functional antithrombin levels.* Methods*. A review of the literature was conducted for studies on prophylaxis and management of VTE in pregnant patients with hereditary AT deficiency. The search involved a number of electronic databases, using combinations of keywords as described in the text. Only English language studies between 1946 and 2015 were included.* Conclusion*. Antithrombin concentrate is indicated in pregnant women with hereditary AT deficiency who develop VTE despite being on therapeutic dose anticoagulation. Expert opinion suggests AT concentrate should be used concomitantly with therapeutic dose anticoagulation. However, further high-quality studies on the dose and duration of treatment in the postpartum period are required. Use of AT concentrate for prophylaxis is controversial and should be based on individual VTE risk stratification.

## 1. Introduction

Antithrombin is a natural anticoagulant that inhibits thrombin (factor IIa), factor Xa, and other serine proteases in the coagulation cascade. As a serine protease inhibitor, its activity is accelerated more than 1000-fold by heparin binding [1]. In the absence of AT, heparin has little effect on anticoagulation [1, 2].

Hereditary AT deficiency is a rare disorder affecting 0.02 to 0.2 percent of the general population [1, 3, 4]. There are several types of hereditary AT deficiency. Type I deficiency involves any genetic mutation that reduces synthesis or other biochemical mechanisms, resulting in decreased serum levels and activity of AT, often defined by less than 70% of plasma AT antigen (immunoassay) and activity (functional assay) [5, 6]. Type II AT deficiency is a qualitative defect in the function of AT affecting primarily the thrombin/Factor Xa and heparin binding sites. As such, it is identified in patients with normal AT antigen but AT activity <70% of normal [6]. Type II AT deficiency can be divided into three subtypes: genetic mutations affecting the reactive site (RS; IIa), heparin binding site (HBS; IIb), and pleiotropic mutations affecting the s1C-s4B region near the RS (IIc) [5, 7]. Most patients with hereditary AT deficiency are heterozygous, since homozygous variants (except for HBS mutations) are thought to be incompatible with life [5, 7–9].

In general, AT deficiency is considered to be a highly thrombogenic condition, with an odds ratio of VTE of 16.3 compared to a nonthrombophilic state [10]. Most (50–90%) untreated individuals with hereditary AT deficiency will suffer thrombosis within their lifetime, even in the absence of additional thrombotic risk factors [11, 12], and are at higher risk in the setting of pregnancy, oral contraceptives, surgery, and trauma [13]. In addition, heparin resistance has been described in some AT deficient patients, leading to inadequate activated partial thromboplastin time (aPTT) prolongation with heparin treatment [14]. The risk of thrombosis depends on the type of AT deficiency and how severely its function or level are affected [15]. The thrombogenicity of type I AT deficiency depends on the residual level of AT activity and/or antigen [7, 9, 16, 17]. Subtype IIa AT deficiency carries the highest risk of thrombosis among the different types of AT deficiency [7]. Although subtype IIb AT deficiency is associated with a lower risk of thrombosis in the general population of heterozygous carriers [18, 19], certain mutations of this subtype carry higher risk than all other types, as observed in patients with the Budapest mutation (L131F) [15, 20]. These founder mutations of L131F and P73L have been associated with high thromboembolic risk in Hungarian and Finnish populations, respectively, [20, 21]. Furthermore, The A384S mutation, common in British and Spanish populations, was found to increase risk of VTE by 9-fold [22]. Genetic analysis of hereditary AT deficiency is necessary for risk stratification of VTE in these patients; however, it is not feasible in day-to-day clinical practice given the multitude of possible mutations [5].

In the general population, pregnant women have a 5-fold higher risk for VTE compared to nonpregnant women [23]. This risk is higher in the postpartum period and may extend for up to 12 weeks [24, 25]. Women with hereditary AT deficiency who are also pregnant are at further risk of VTE during pregnancy, although estimates are highly variable [26, 27]. In one cohort of pregnant women with inherited thrombophilia, 7/50 (14%; 95% CI 8–28%) AT deficient women developed deep vein thrombosis (DVT) during pregnancy compared to 0.2% in the general population [26]. This risk was shown to be higher in the postpartum period (11/39 28%; 95% CI 30–36%) [26]. However, in a subsequent study, the effect of pregnancy and other acquired risks of VTE did not alter their annual risk of VTE (1.7%/year), which was attributed to the use of anticoagulation in these situations [11]. Furthermore, these rates were deemed lower in a more recent systematic review of pregnant women with thrombophilia [28]. The odds ratio of developing VTE was 4.69 (95% CI 1.30–16.96) in AT deficient women compared to the general population [26]. Women with AT deficiency are also at increased risk of pregnancy loss despite use of prophylactic heparin [29]. In addition, maternal AT deficiency is associated with higher risks of intrauterine growth restriction, placental abruption, preeclampsia, and intrapartum fetal distress [13].

The high risk of thrombotic events in pregnant patients with hereditary AT deficiency has prompted a recommendation from a number of societies such as the Royal College of Obstetricians and Gynecologists (RCOG) and the American Congress of Obstetricians and Gynecologists (ACOG) supporting prophylactic low molecular weight heparin (LMWH) use in hereditary AT deficient patients with a personal or family history of VTE [30, 31]. However, patients may still develop VTE despite being on LMWH. In selected patients with previous pregnancy-associated VTE, AT concentrates may be considered for secondary prophylaxis of pregnancy-induced VTE. Pregnant patients with hereditary AT deficiency who develop VTE during pregnancy are often treated with both anticoagulants and AT concentrate. There are no guidelines regarding management of acute VTE in pregnant patients with AT deficiency, and recommendations are based on expert opinion. There is little data available on the appropriate timing of initiation, dosing, and duration of AT concentrate during pregnancy and the postpartum period. We present a clinical case of a pregnant woman with hereditary AT deficiency who developed DVT and was treated with AT concentrate and present a review of the literature regarding the use of AT concentrate in pregnant women with hereditary AT deficiency.

## 2. Case Presentation

The patient is a woman who was initially diagnosed at the age of 22 with inherited type I AT deficiency as part of a family screen, with immunological AT level of 0.52 U·mL^−1^ (reference range 0.80–1.20 U·mL^−1^) and functional AT level 0.49 U·mL^−1^ (reference range 0.77–1.25 U·mL^−1^) (Coamatic Antithrombin assay from Chromogenix). Molecular genetic testing was not performed. The patient's mother and two sisters were also identified as having AT deficiency. Her mother had a history of unprovoked DVT and pulmonary embolism (PE) at age 50. One of her sisters was the index case, having suffered a fatal PE six months postpartum at the age of 18. Her younger sister (immunological AT 0.44 U·mL^−1^, functional AT 0.41 U·mL^−1^) experienced a cerebral vein thrombosis 34 weeks into her first pregnancy despite being on therapeutic dose LMWH (dalteparin 200 units·kg^−1^ daily). This sister was treated with AT concentrate through the remainder of her pregnancy and until six weeks postpartum as described previously [32]. A summary of the patient's family history is provided in Table 1. The patient had a history of thromboembolic events, including a DVT and PE at age 25. Apart from her inherited AT deficiency, she did not have any other inherited or acquired risk factors for thrombosis. Her initial DVT and PE were managed with warfarin until age 36, when she was switched to rivaroxaban and later developed amaurosis fugax. At that point, she was treated with warfarin.

At the age of 37 (July 2015), the patient was found to be pregnant and, in consultation with her hematologist, was transitioned from warfarin to unmonitored therapeutic dose LMWH (tinzaparin) at 175 units·kg^−1^ (12,000 units) daily. One week later she presented with a one-day history of left leg pain, and an ultrasound, compared to previously normal imaging in January 2015, confirmed the presence of a new proximal DVT involving the superficial femoral vein, popliteal vein, and the trifurcation. Laboratory investigations upon presentation included hemoglobin 114 g·L^−1^; platelet count 194 × 10^9^ L^−1^; creatinine 61 mmol L^−1^; INR 1.0; and aPTT 26 seconds. Her functional AT level was 0.58 U·mL^−1^ (reference range 0.77–1.25 U·mL^−1^). Pelvic ultrasound showed an intrauterine pregnancy with an estimated gestational age of six weeks.

The patient was admitted to hospital and switched to subcutaneous LMWH (dalteparin) at 200 units·kg^−1^ (7,500 units every 12 hours). Her weight was 69.1 kg. She received plasma-derived AT concentrate (Baxter) intravenously initially at a dose of 3,000 units to a target trough AT level of 1.20 U·mL^−1^. Functional AT levels were measured prior to each subsequent daily dose of AT, which were adjusted accordingly until her trough functional AT levels were stable between 0.80 and 1.20 U·mL^−1^ on 5,000 units intravenously every other day.

Approximately one week after her initial presentation, the pregnancy was deemed unviable and the patient consented to aspiration and curettage. The patient was switched to intravenous unfractionated heparin to achieve a target aPTT of 60–85 seconds preoperatively. She also received 3,000 units of AT concentrate on the day before the procedure, 5,000 units after procedure, and then 5,000 units every other day for a total of six weeks postpartum. On the day following the procedure, she was restarted on tinzaparin 175 units·kg^−1^ (12,000 units) daily subcutaneously as a bridge to warfarin to achieve a target INR of 2.0 to 3.0. She was discharged from hospital after a total of 12 days, with the AT concentrate administered on an outpatient basis. She did well on the combination therapy with no episodes of bleeding or thrombosis. The AT concentrate was discontinued after six weeks.

## 3. Discussion

Hereditary AT deficiency is a rare disorder which may have adverse effects on the outcome of pregnancy in affected patients. In this case report, a woman with known hereditary AT deficiency presented with DVT during her pregnancy despite therapeutic anticoagulation. Given her history of previous VTE and current pregnancy, a decision was made to manage her DVT aggressively using a combination of anticoagulation and AT replacement. To explore other patients with similar presentations we undertook a literature search of multiple electronic databases (including EMBASE, PubMed/MEDLINE) to look for other similar cases. Different combinations of the following keywords: “hereditary,” “antithrombin deficiency,” “ATIII,” “pregnancy,” “antithrombin concentrate,” “case report/series,” and “clinical study” were used in the search. There were no date limits on the search which was conducted in December 2015. The search returned 439 publications, with 25 studies [32–56] meeting the following inclusion criteria: adults with hereditary AT deficiency and requiring AT concentrate during pregnancy for either therapeutic (if developed VTE during pregnancy) or prophylactic (no VTE developed during pregnancy) indications. Of these studies, 23 cases and 24 pregnancies were reviewed where AT concentrate was used to treat a VTE during pregnancy (Table 2); 10 cases were reviewed where AT concentrate was used as prophylaxis for VTE in the antepartum period (Table 3). Two studies were reviewed in an integrated analysis which included 21 patients who received AT concentrate as prophylaxis around the time of delivery [33]. An additional 20 cases of peripartum prophylactic AT concentrate use were reviewed in other case reports/series.

### 3.1. Characteristics of AT Concentrate

Two types of AT concentrates are commercially available. Antithrombin concentrate derived from pooled human plasma is indicated in the treatment and prevention of VTE in patients with hereditary AT deficiency whose AT activity is less than 70%. Recombinant human AT concentrate, derived from the milk of genetically engineered goats, is approved in the United States for the prevention of perioperative and peripartum thromboembolic events in hereditary AT deficient patients. The most common (>5%) adverse reactions from AT concentrate infusion include hemorrhage (intra-abdominal, hemarthrosis, and postprocedural), and infusion site reaction. The risk of transmission of infectious agents with human plasma-derived AT concentrate is low, and serious adverse events have not been reported in the literature.

### 3.2. Indications of AT Concentrate

The two most common indications for AT concentrate in pregnant women with hereditary AT deficiency are for the management of acute VTE during pregnancy despite therapeutic anticoagulation and for prophylaxis in the antepartum, peripartum, and/or postpartum periods. The indication is less clear in women who develop VTE while receiving prophylactic dose anticoagulation, who can potentially be managed by increasing the dose of anticoagulation to therapeutic levels. AT concentrate can be given concurrently with anticoagulation in select, high risk patients including those with catastrophic or severe thrombotic events. However, there is no recommendation from the ACOG or American College of Chest Physicians (ACCP) guidelines on how to appropriately dose and monitor AT concentrate during pregnancy, or the optimal duration of AT concentrate to continue postpartum [30, 57–60].

### 3.3. Therapeutic Use of AT Concentrate in Pregnancy

AT concentrate is indicated in pregnant women with hereditary AT deficiency who develop VTE despite being on therapeutic dose anticoagulation. For human plasma-derived AT (Thrombate III), the loading dose is calculated using the following equation:

(1) Dose of AT concentrate IU=Target%−Baseline% AT level×weight kg1.4% kg IU−1.

Functional AT activity should be measured before, 20 minutes (peak) and 12 hours after the loading infusion, and preceding the next dose (trough). This will help determine the required maintenance dose and interval between doses, which is often adjusted to achieve functional AT activity of 80–120%. Subsequent monitoring can be done before dose. As a rule of thumb, the maintenance dose is often 60% of the loading dose, given every two to three days. The biological half-life of human plasma-derived AT concentrate is 2.5 days based on immunologic assays and 3.8 days based on functional assays of AT. In some clinical situations (e.g., after surgery, hemorrhage, acute thrombosis) the half-life of AT is reported to be shortened.

23 cases and 24 pregnancies were reviewed where AT concentrate was used therapeutically (Table 2). From our review of the literature, the dose of AT used in the different studies ranged from 24 to 65 IU kg^−1^, two or three times a week, and was titrated to a targeted, trough AT activity of 80–120%. The duration of AT replacement is uncertain. In most studies, treatment was initiated at the time of the VTE and continued until delivery, or for several weeks postpartum. In one report, warfarin was restarted in the second trimester and AT was given for a short period only, until the INR reached a therapeutic range [51]. Due to the high risk of VTE during the six-week postpartum period, therapeutic anticoagulation (either warfarin or LMWH) should be continued. AT concentrate is usually continued for six to nine days after delivery (Table 2). However, in our case, given the patient's family history of fatal PE during the postpartum period, we continued AT concentrate empirically for an additional six weeks.

Given the rarity of AT deficiency, large clinical trials studying the efficacy of AT concentrates in pregnant patients are lacking. However, based on limited, small studies of AT deficient patients undergoing surgery or giving birth, AT concentrate was shown to lower rates of VTE when anticoagulation was held during the perioperative and peripartum periods [41, 49, 61]. Other cases reported in the literature suggest that pregnant women with hereditary AT deficiency who receive therapeutic AT concentrate during pregnancy might have low rates of recurrent thrombosis and bleeding [38]. In this review, across the 23 pregnant patients (a total of 24 pregnancies), 14 (58%) pregnancies had term deliveries with favorable outcomes for the mother and newborn. Six (25%) pregnancies resulted in pregnancy loss despite AT concentrate. Two pregnancies resulted in miscarriage, and four underwent therapeutic/induced abortion [34, 38, 55]. In one study, a pregnant woman was found to have an iliofemoral DVT at gestational week 28, and, despite receiving therapeutic dose anticoagulation, AT concentrate 2000 IU daily for three days, and an IVC filter, developed PE [39]. All of the 23 patients were treated concomitantly with therapeutic anticoagulation (Table 2). Based on these reports, AT concentrate appears to be effective in managing acute VTE and preventing VTE recurrence during pregnancy and in the postpartum period. Among patients who developed VTE during pregnancy and completed a course of AT replacement, one patient developed VTE on the first postpartum day [42], and the second patient at 4.5 weeks postpartum [49] requiring reinitiation of AT concentrate. This signifies the persistent, increased risk of thrombosis during that period.

The Robertson et al. (2006) meta-analysis examined the risk of VTE and adverse outcomes associated with thrombophilia in pregnancy, as well as the effectiveness of prophylactic measures [28]. They reported increased VTE risk in the subgroup analysis of patients with AT deficiency who did not receive AT concentrate [62–64], without adverse outcome on pregnancy [65–67]. Beside the small population size, they noted lack of randomized control trials for antithrombotic interventions in this patient population. In our review, we have shown that despite most of the cases being on anticoagulation during pregnancy they still developed VTE and related complications requiring concurrent administration of AT concentrate.

### 3.4. Prophylactic Use of AT Concentrate in Pregnancy

The use of AT concentrate for prophylaxis in pregnant women with hereditary AT deficiency is not as well studied. The prophylactic use of AT concentrate can be described as either primary or secondary and can be utilized in any of the antepartum, peripartum, or postpartum periods. In most studies, prophylaxis is used in patients with an extensive personal history of VTE or a history of repeated, unexplained pregnancy loss. We reviewed 10 cases where AT concentrate was used as antepartum prophylaxis for thrombotic events (Table 3). The triggers for initiation of AT concentrate during the antepartum period were not consistent among the studies reviewed. Some reports initiated AT concentrate as soon as a pregnancy was confirmed and continued until delivery [35], or if initiation of therapeutic LMWH during first trimester was deemed ineffective (subtherapeutic anti-Xa levels) [36]. The dose and frequency of administration of AT concentrate were used in the same manner as treatment of VTE, which targets a trough AT level of 80–120%.

In terms of the peripartum period, a recent integrated analysis of two prospective studies concluded that AT concentrate is safe and effective in reducing VTE during the peripartum period if administered over an average period of four days [33, 37, 41]. Of the 21 included patients, 19 had a previous personal history of VTE. Beyond seven days, one patient developed DVT 11 days and one developed PE 14 days after AT concentrate discontinuation; both patients were also receiving prophylactic dose anticoagulation. Four other patients developed severe adverse events several weeks after the discontinuation of AT concentrate (e.g., intra-abdominal bleeding, anterior rectus muscle hematoma, sepsis, and pyrexia) all of whom resolved with appropriate management. An additional 20 cases were described in other studies [35, 36, 38, 47–49, 51, 53–56]. Although most of these cases started AT concentrate 24 hours before the time of expected delivery, they did not have a consistent initiation or discontinuation timeline. Three cases who developed VTE during pregnancy were managed with therapeutic anticoagulation and were later started on AT concentrate as prophylaxis around delivery and continued for six days postpartum [38, 48]. The dose of AT used in these studies ranged from 30 to 93 IU kg^−1^ daily and was often titrated to a targeted AT activity of 80–120%. In all of the additional 20 cases, there was a successful outcome for the mother and newborn, except for one mother who suffered from disseminated intravascular coagulation [56]. AT concentrate has also been administered around the time of therapeutic abortions [55].

Few reports utilized AT concentrate for primary prophylaxis. In one study, two patients were started on primary prophylaxis with AT concentrate for significant family histories of VTE and/or extensive personal history of miscarriages [34]. Despite receiving AT concentrate, pregnancy loss was reported in both cases. Pregnancy loss despite AT replacement was reported in a second case report [47].

## 4. Summary

*Therapeutic AT Concentrate Use*

- Pregnant women with hereditary AT deficiency and a history of VTE should receive therapeutic anticoagulation appropriate for their term of pregnancy.
- Pregnant women with hereditary AT deficiency who develop VTE despite being on prophylactic dose anticoagulation would benefit from switching to a therapeutic dose of anticoagulation. In this situation, concomitant administration of AT concentrate may be considered, but more research is required.
- Pregnant women with hereditary AT deficiency who develop VTE despite being on therapeutic dose of anticoagulation seem to benefit from the concomitant initiation of AT concentrate. It can be started with a loading dose between 30 and 50 IU kg^−1^ with frequent monitoring until a trough level of AT activity of 80–120%, at which time the dose can be used for maintenance. The maintenance dose is often given every two to three days if adequate trough levels are achieved. The duration of treatment is not well established, but we recommend continuation until there is resolution of signs and symptoms of VTE. Therapeutic anticoagulation seems to have a beneficial effect when continued into the postpartum period for six weeks; however, some expert opinion might recommend extension to 12 weeks. The role and duration of AT concentrate in the puerperium period is unclear, and more studies are needed to answer these uncertainties. However, AT concentrate would seldom be continued for more than a few days after delivery, once appropriate therapeutic anticoagulation has been started.

*Prophylactic AT Concentrate Use*

- No clear guidelines are available to guide appropriate use of AT concentrate in pregnant women with hereditary AT deficiency in the absence of VTE.
- There is no clear benefit from the use of AT concentrate as primary prophylaxis against VTE in pregnant patients with hereditary AT deficiency. Evidence is lacking to justify its use, as associated costs and risks may outweigh anticipated benefits.
- Use of AT concentrate for secondary prophylaxis in the antepartum period requires individualized thrombotic risk assessment including a patient's previous history of VTE (particularly a history of unusual site VTE, VTE during pregnancy, or recurrent VTE despite therapeutic dose anticoagulation) and consideration of the type of AT deficiency must be considered.
- In the* postpartum period*, AT concentrate could be considered in a hereditary AT deficient pregnant woman who had previous history of VTE, especially during a previous pregnancy.

## Figures and Tables

**Table 1:** Family History of venous thromboembolic events of the index case.

Relationship	Age at diagnosis	Type of AT deficiency	Other inherited or acquired risk factors	History of VTE	Management	Outcome
Mother	50	1	Smoking	DVT-PE	N/A	VTE resolved

Sister 1	18	1	Postpartum at time of death	Fatal PE	N/A	Fatal

Sister 2	25	1	Pregnant at time of VTE	Cerebral vein thrombosis	LMWH + AT concentrate	VTE resolved, successful delivery at 34 weeks

Index case	22	1	None	DVT & PE at age of 25	Warfarin until age of 36	Developed amaurosis fugax, switched to rivaroxaban
				DVT during pregnancy at age 37	Initially on tinzaparin (for pregnancy) but switched to dalteparin and AT concentrate	Miscarriage at GW 7, received D & C after which continued warfarin and AT concentrate × 6 weeks

AT: antithrombin, D & C: dilatation and curettage, DVT: deep vein thrombosis, gw: gestational week, LMWH: low molecular weight heparin, and PE: pulmonary embolus.

**Table 2:** AT concentrate for treatment of VTE in patients with hereditary AT deficiency and pregnancy.

Study	Study design	Case	Thrombotic condition	Previous anticoagulation	AT Peripartum prophylaxis	Type and dosage of AT used	Concurrent anticoagulation	AT concentrate initiation/duration	Goal of AT levels	Outcome
Ilonczai et al. 2015	Retrospective	Pt1	Bilateral DVT at gw 7	Yes	No	N/A	Therapeutic dose LMWH	VTE event to miscarriage	80–120%	Miscarriage at gw8
		Pt2	PE at gw 7	Yes	50 IU × 2 days	2500 3tw		VTE event to delivery		Healthy M and N

Rogenhofer et al. (2014)	Retrospective	Pt1G1	DVT Lt arm at gw 12	Yes	N/A	1500 3tw	Weight adjusted prophylactic dosage LMWH	VTE event to delivery	N/A	Healthy M and N
		Pt1G2	Sinus thrombosis gw 11			1500 Q3D				Healthy M and N

Bramham et al. (2013)	Retrospective	Pt1	Sinus thrombosis gw 11	No (new Dx)	50 IU/Kg	3000 IU alternate days	Therapeutic UFH infusion	VTE event to delivery	>80%	Healthy M and N
		Pt2	Sinus thrombosis gw 9	Yes	50 IU/Kg	3000 IU alternate days		N/A		Healthy M and N
		Pt3	DVT at 28 gw	Yes	50 IU/Kg	2000 IU OD × 3 days after IVC filter insertion		3 days after IVC filter insertion		M:PE after IVC filter insertion. N: required NICU

James et al. (2013)	Prospective	Pt1	DVT, PE at gw 20	No (new Dx)	N/A	Plasma-derived AT concentrate (Thrombate III) Loading dose (in units) = (120% − basal% normal) × wt in kg/1.4 Maintenance dose = 60% of loading dose	Therapeutic UFH	Prior delivery to 6 days postpartum	After LD, 80% Maintenance dose, 70–120%	Healthy M and N
		Pt2	DVT early in pregnancy	Yes				Preeclampsia early labor		Healthy M and N
		Pt3	DVT left leg at gw 8	Yes				VTE event to delivery		Healthy M and N
		Pt4	PE at gw 12	Yes				VTE event to delivery		Healthy M and N
		Pt5	DVT at gw 9	Yes				VTE to abortion		Therapeutic abortion
		Pt6	PE at gw 12	Yes				VTE to abortion		Therapeutic abortion

Tanimura et al. (2012)	Case report	Pt1	DVT at gw7, HIT	No (new Dx)	1500 IU for 2 days	3000 IU loading then 1500 IU 2tw	UFH then switch to argatroban	VTE event to delivery	>70%	Healthy M and N

Sharpe et al. (2011)	Case report	Pt1	SVT at gw 34	Yes	3000 IU for 3 days	Plasma-derived AT concentrate: 3000 daily for 5 days then alternate 2000 IU and 3000 IU continued for 6 weeks postpartum	UFH IV	VTE event to delivery	100%	Healthy M and N

Hidaka et al. (2008)	Case report	Pt1	LE DVT at gw 24	No	3000 IU for 1 day	3000 IU 2-3x per week	UFH IV + IVC filter	VTE event to delivery	>70%	M: progression of DVT & developed PE

Alguel et al. (2007)	Case report	Pt1	Pathological flow in umbilical artery at gw 35	Yes	2000 IU for 7 days	6000 IU × 1 dose 2000 IU × 6 days postpartum	Therapeutic LMWH	VTE event to 6 days postpartum	>70%	Healthy M and N

Shiozaki et al. (1993)	Case report	Pt1	DVT in early pregnancy, recurrence at gw 37	Yes	2000 IU × 1 day	2000 IU first dose, then 1000 IU once weekly	Therapeutic LMWH	Gw 37 to 9 days postpartum	>80%	Healthy M and N

Kario et al. (1992)	Case report	Pt1	DVT at gw 6	Yes	3000 IU × 1 day	Not indicated	UFH IV	Gw 6, not indicated Restarted Gw 34–40 (2° prophylaxis)	>80%	Healthy M and N

Menache et al. (1990)	PROBE	Pt1	DVT at gw 10	Yes	Yes	Plasma-derived AT-III concentrate Total 14,563 IU for 5 d Total 17,355 IU for 7 d	UFH IV	Gw 10-11 & postpartum (5 days)	80–120%	Healthy M and N
		Pt2	DVT at gw 14	Yes	No			GW 14-15 without postpartum prophylaxis		M: developed Pelvic and vena cava DVT 4.5 weeks after delivery

Hellgren et al. (1982)	Case series	Pt1	DVT at gw 13	Yes	N/A	Plasma-derived AT-III concentrate Total 28500 IU for 10 d Total 7500 IU for 4 d 8000 IU for 1 dose	UFH IV	Gw 13, abortion	80–120%	Therapeutic abortion
		Pt2	DVT at gw 6					Gw 6, miscarriage		Miscarriage
		Pt3	DVT early in pregnancy					A single dose		Therapeutic abortion

AT: antithrombin, DVT: deep vein thrombosis, Dx: diagnosis, gw: gestational week, HIT: heparin-induced thrombocytopenia, IU: international units, IVC: inferior vena cava, LE: lower extremity, LMWH: low molecular weight heparin, M: mother, N/A: not available or not specified, N: newborn, OD: daily, PE: pulmonary embolus, PROBE: prospective randomized open blinded end-point, Pt: patient, Q3D: every 3 days, UFH: unfractionated heparin, wt: weight, 2tw: two times per week, and 3tw: three times per week.

**Table 3:** Thrombosis medical profile and *antepartum prophylactic* use of AT concentrate in pregnant patients with hereditary antithrombin deficiency.

Study	Study design	Number of cases	Prophylactic condition and numbers of cases		Dosage of AT used	Concurrent anticoagulation	AT concentrate initiation/duration	Goal of AT levels	Outcome
			Primary	Secondary					
Ilonczai et al. 2015	Retrospective	4	2	2	1000–2500 2tw-3tw	LMWH	Primary, gw 11–28; gw 20–36 Secondary, gw 6–13; gw 25–29	80–120%	Miscarriage gw 28 Healthy M and N Miscarriage gw 3 Healthy M and N

Rogenhofer et al. (2014)	Retrospective	4	1	3	1000 2tw- 2500 IU 3tw	LMWH	Primary, 3 days as initiation of LMWH at early pregnancy to delivery Secondary, gw 6, 7, 34 to delivery	N/A	Healthy M and N

Pascual et al. (2014)	Case report	1	—	1	3000 IU 3tw	LMWH, UFH then warfarin at 14 wk	gw 4–15 and 11 days peripartum	>50%	Healthy M and N

Yamada et al. (2001)	Case report	1	—	1	3000–6000 IU weekly	N/A	gw 5–38	80–90%	Healthy M and N

AT: antithrombin, gw: gestational week, IU: international units, LMWH: low molecular weight heparin, M: mother, N/A: not available or not specified, N: newborn, UFH: unfractionated heparin, gw: gestational week, IU: international units, IVC: inferior vena cava, UFH: unfractionated heparin, 2tw: two times per week, and 3tw: three times per week.

## Abbreviations

ACCP: American College of Chest Physicians
ACOG: American Congress of Obstetricians and Gynecologists
AT: Antithrombin
DVT: Deep vein thrombosis
HBS: Heparin binding site
IU: International units
LMWH: Low molecular weight heparin
PE: Pulmonary embolism
RCOG: Royal College of Obstetricians and Gynecologists
RS: Reactive site
VTE: Venous thromboembolism.
